# Eu-Chelate Polystyrene Microsphere-Based Lateral Flow Immunoassay Platform for hs-CRP Detection

**DOI:** 10.3390/bios12110977

**Published:** 2022-11-07

**Authors:** Birui Jin, Zhiguo Du, Chuyao Zhang, Zhao Yu, Xuemin Wang, Jie Hu, Zedong Li

**Affiliations:** 1 School of Materials and Chemical Engineering, Xi’an Technological University, Xi’an 710021, China; 2 Bioinspired Engineering and Biomechanics Center (BEBC), Xi’an Jiaotong University, Xi’an 710049, China; 3 Xi’an Thermal Power Research Institute Co., Ltd., Xi’an 710054, China; 4 The Key Laboratory of Biomedical Information Engineering of Ministry of Education, School of Life Science and Technology, Xi’an Jiaotong University, Xi’an 710049, China; 5 Department of Radiotherapy Hospital Unit Radiation Therapy, Shaanxi Provincial Tumor Hospital, Xi’an 710061, China; 6 Suzhou DiYinAn Biotech Co., Ltd., Suzhou Innovation Center for Life Science and Technology, Suzhou 215129, China

**Keywords:** fluorescence analysis, sensitivity enhancement, paper microfluidics, hs-CRP detection

## Abstract

**Inflammation** caused by viral or bacterial infection is a major threat to human health globally. Blood C-reactive protein (CRP) has been proven to be a sensitive indicator for the occurrence and development of inflammation. Furthermore, a tiny change of blood CRP concentration may portend chronic diseases; therefore, high-sensitivity CRP (hs-CRP) detection in a quantitative, rapid, user-friendly, and low-cost manner is highly demanded. In this paper, we developed a europium-chelate polystyrene microsphere (EuPSM)-based lateral flow immunoassay (LFIA) integrating with a benchtop fluorescence analyzer for hs-CRP detection. The optimization of the EuPSM-based LFIA was implemented through adjusting the antibody density on EuPSM from 100% to 60% of the saturated density. Finally, the limit of detection of 0.76 pg/mL and detection range of 0.025–250 ng/mL were obtained. Moreover, the clinical application capability of the proposed platform was validated through detecting CRP in clinical serum samples, showing high consistency with the results obtained from the clinical standard method. Hence, the proposed EuPSM-based LFIA has been verified to be well suitable for hs-CRP detection, while also showing great applicability for sensitively and rapidly detecting other biomarkers.

## 1. Introduction

Inflammation caused by viral or bacterial infection is a major threat to human health globally. For example, hepatitis and pneumonia are responsible for nearly 10 million deaths a year [1]. Blood C-reactive protein (CRP) has been proven to be a sensitive indicator for the occurrence and development of inflammation [2]. Furthermore, a tiny change of blood CRP concentration may portend chronic diseases, such as cardiovascular diseases, peripheral vascular diseases, and neonatal sepsis; therefore, high-sensitivity CRP (hs-CRP) detection at a level of 1 ng/mL is demanded [3,4]. Clinically, CRP is detected by the enzyme-linked immunosorbent assay (ELISA) and chemiluminescent immunoassay, exhibiting high accuracy and sensitivity [5,6] but requiring professional operators and long testing time. The ideal hs-CRP detection should be implemented outside of central labs with features of quantification, rapidity, user friendliness, and low cost [7]. 

Lateral flow immunoassay (LFIA), as the most widely used biosensor, was employed to realize the detection of many disease markers due to its characteristics of rapidity, cheapness, and ease of operation [8,9,10]. However, the low sensitivity of conventional LFIA usually limits its application in hs-CRP detection, due to the weak colorimetric signal generated by conventional nanoparticles (e.g., AuNPs [10], AgNPs [11]). Nowadays, layered transition-metal dichalcogenide (TMD) materials (e.g., VS_2_ nanosheet [12] or MoS_2_ nanosheet [13]) have been creatively employed in LFIA development as colorimetric signal reporters, due to their highly specific surface areas and good protein adsorption ability [14]. Although these TMD-LFIAs exhibit good performance, their application is still limited by the difficulty of quantitative detection. Alternatively, fluorescence nanoparticles (e.g., fluorophores, quantum dots (QDs), and upconversion nanoparticles (UCNPs)) were employed, where the fluorescence signal can be directly quantified by photoelectric sensors [15,16]. However, fluorophores-based LFIAs suffer from a relatively limited sensitivity, due to narrow Stokes shift (20–30 nm), photobleaching, and low emission intensity [17]. The excellent fluorescence properties of QDs and UCNPs support the development of optimized LFIA [18,19], but unstable and complex preparation and modification processes limit their large-scale application [17]. Recently, the europium-chelate polystyrene microsphere (EuPSM) possesses a longer fluorescence lifetime (µs) than traditional fluorophores (ns), allowing signal to be collected beyond the lifetime of background fluorescence [20]. Moreover, its long Stokes shift ensures that incident light from the excitation source (λ~330–340 nm) does not interfere with collection of light by the detector (λ~610–620 nm) [21]. These attractive traits create new opportunities in constructing high-sensitivity LFIAs. Considering that the size of EuPSM is usually approximately 250–300 µm, much larger than that of traditional fluorescence nanoparticles, it may significantly increase the number of binding sites on the surface of the microsphere. Thus, the amplified fluorescence signal may be obtained through optimizing modified probe (e.g., antibody) density [22,23]. The femtosecond pulsed UV radiation source-based photonic immobilization technique (PIT) can effectively improve the binding ability of the antibody, while reducing the degeneration of biomolecules affected by UV light [24], which is expected to achieve the regulation of probe density but may be limited by the high requirements of operation and equipment. Hence, optimization of EuPSM-based LFIA based on simpler and more effective ways is still needed to meet the requirements of ultra-sensitive POCT platform.

In this work, we developed an optimized EuPSM-based LFIA coupled with a portable fluorescence analyzer for ultra-sensitive hs-CRP detection. Firstly, the antibody (anti-CRP Ab8#) density on EuPSM was systematically investigated from 90% to 10%. Then, the concentration of EuPSM-anti-CRP Ab8# was optimized. Finally, a portable fluorescence analyzer with the capability of scanning the fluorescence intensity on the LFIA point by point was developed. The proposed LFIA has been verified to be suitable for hs-CRP detection with ultra-high detection sensitivity and a wide detection range. The proposed platform also shows huge potential for sensitively and rapidly detecting other biomarkers.

## 2. Materials and Methods

### 2.1. Materials

Eu(DBM)_3_phen and carboxylic PSM were purchased from Bangs Laboratories, Inc. (Fishers, IN, USA). Trizma base (Tris), 1-ethyl-3-(3-dimethylaminopropyL)-carbodiimide hydrochloride (EDC), and bovine serum albumin (BSA) were purchased from Sigma Aldrich. N-hydroxysulfosuccinimide sodium salt (Sulfo-NHS) and glycine (Gly) were obtained from Aladdin. Ethanolamine, ethylenediamine tetraacetic acid disodium salt dihydrate (EDTA), sodium dihydrogen phosphate dihydrate (NaH_2_PO_4_·2H_2_O), disodium hydrogen phosphate (Na_2_HPO_4_), and sodium chloride (NaCl) were obtained from China National Medicines Corporation Ltd. MES monohydrate (MES) and Hepes were purchased from Beijing Solarbio Technology Co., Ltd. (Beijing, China). Tween 20 and d-trehalose were obtained from Shanghai Macklin Biochemical Technology Co. (Shanghai, China). Surfactant S9 was obtained from Jiangsu Pengxin Biochemical., Ltd. (Nantong, China). Antibodies (anti-CRP Ab8#, anti-CRP Ab7#, Goat anti-mouse IgG) and CRP antigen were purchased from Fapon Novus Limited Partnership. The backing pad (80 × 300 mm), sample pad (SB08, 15 × 300 mm), absorbent pad (H-1, 30 mm × 300 mm), immersing pad (8965, 10 mm × 300 mm), and nitrocellulose membrane (NC membrane, Sartorius CN 95, 25 × 300 mm) were obtained from Shanghai Jiening Biological Technology Co., Ltd. (Shanghai, China). All reagents were of analytical grade and used without any purification.

### 2.2. Preparation of EuPSM

The EuPSM was synthesized by encapsulating Eu(DBM)_3_phen into monodisperse PSMs according to published procedure [17]. Briefly, 150 mg of Eu(DBM)_3_phen was dissolved in 10 mL of CH_2_Cl_2_ and mixed with 100 mL of carboxylic PSM (3%, *w*/*v*, dispersed in 0.25% of SDS). The mixture was added to a 200 mL of Erlenmeyer flask and sonicated for 15 min to form a uniform suspension. The product was stirred at room temperature for 24 h, and then heating at 50 °C in a water bath over night to completely evaporate the CH_2_Cl_2_. The product was washed three times with ethanol, dispersed in water (1% *w*/*v*), and stored at 4 °C.

### 2.3. Antibody Modification on EuPSM

A total of 100 μL of EuPSM was washed with MES buffer (50 mM, pH = 6.0) four times. The mixture was placed in a shaker (37 °C, 220 rpm) for 45 min after adding 12.5 μL, 100 mg/mL of EDC and 12.5 μL, 100 mg/mL of sulfo-NHS. After washing with MES buffer by three times, 500 µL of MES buffer and 12.6 µL of anti-CRP Ab8# were added into the as-prepared mixture in sequence and shook for 2 h. Then, the mixture was washed with ultra-pure water twice, and then 200 µL of blocking solution (5 µL/mL of ethanolamine and 20 mg/mL of BSA) was added and shook for 30 min. Finally, after washing with ultra-pure water twice, the mixture was re-dispersed in 200 µL of ultra-pure water and stored at 4 °C for use.

### 2.4. Preparation of EuPSM-LFIA

The EuPSM-LFIA was prepared following the protocol [16,25]. An amount of 15 µL of anti-CRP Ab8#@ EuPSM was diluted by ten times and sprayed evenly onto the immersing pad using an XYZ platform dispenser (Shanghai Jinbiao, HM 3035). Then, the dried immersing pad, absorbent pad, sample pad, and NC membrane were pasted on a backing pad with 1 mm overlap between each two adjacent pads. After spraying goat anti-mouse IgG (as C-line) and anti-CRP Ab7# (as T-line) on the NC membrane, the as-prepared LFIA was dried at 37 °C for more than 2 h. Finally, the as-prepared pads were cut into strips with a width of 3.9 mm using a programmable shear (SATA, ZQ-2002).

### 2.5. Optimization of the Running Buffer and Antibody Concentration on T-Line and C-Line

The component of the running buffer can affect the signal intensity and signal-to-noise ratio. To optimize the running buffer, several basic buffers (ultra-pure water, PBS, Tris-HCl, and Hepes) were tested. Furthermore, additional reagents (i.e., NaCl, Tween 20, BSA) were added into the basic buffer to improve the specific binding between antigen and antibody and prevent non-specific binding on the NC membrane. Through optimization, the optimal running buffer containing Hepes (0.1 M, pH = 8.0), NaCl (0.5% *w*/*v*), Tween 20 (5% *v*/*v*), and BSA (5% *w*/*v*) was obtained. The antibody concentration modified on T-line and C-line can affect its capture capability and signal intensity. After optimization, 1 mg/mL of anti-CRP Ab7# and 1 mg/mL of goat anti-mouse IgG were used as standard dosages for T-line and C-line.

### 2.6. Detection of CRP Samples

All the standard CRP samples were diluted using the optimized running buffer to prepare gradient concentrations (0.01, 0.025, 0.05, 0.25, 0.5, 2.5, 5, 25, 75, 125, 150, 250, 350, 500 ng/mL) for later use. During detection, 100 μL of the sample solution was dripped into the sample area, and flowed through the detection area, where the reaction occurred at the T-line and C-line. The results were read by a smartphone (darkroom condition, 350 nm excitation light, 4 s of exposure time) and a portable fluorescence analyzer, respectively. We repeated this three times to obtain the average values and standard deviations. Particularly, the detection for blank detection (0 ng/mL) was repeated 10 times. The limit of detection (LOD) was calculated according to the equation S/N = 3.

### 2.7. Detection of Clinical Samples 

To further demonstrate the practical ability of the developed EuPSM-LFIA platform, clinical serum samples from Shanghai Chest Hospital were used as real samples. For all samples, the standard concentrations were detected by the chemiluminescent immunoassay method (Mindray Cal8000). All samples were pretreated with a dilution of 500 times using the running buffer.

## 3. Results and Discussion

### 3.1. Optimization of Modified Antibodies on EuPSM for High-Sensitivity LFIA

**Figure 1:**
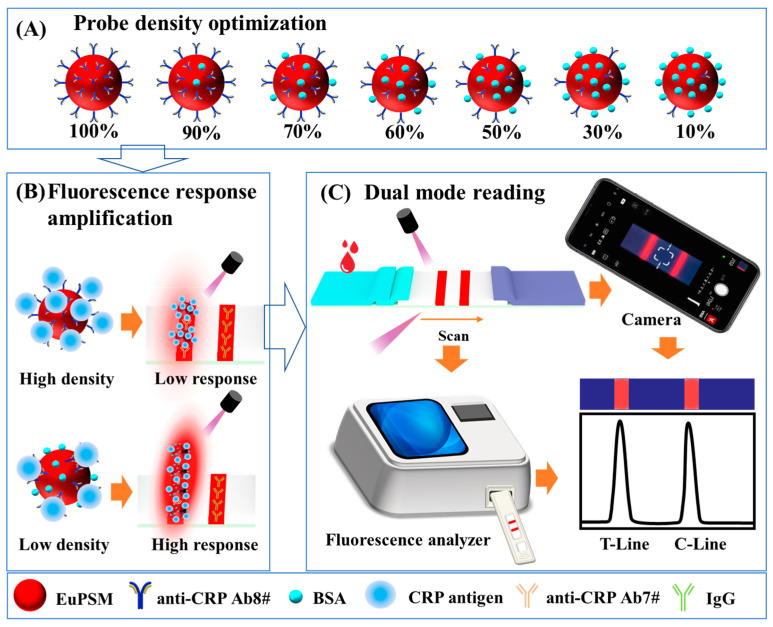
Schematic illustration of Eu-chelate polystyrene microsphere-based lateral flow immunoassay for highly sensitive C-reaction protein detection. (**A**) The probe density optimization on Eu-chelate polystyrene microsphere. (**B**) Schematic showing amplified fluorescence signal on lateral flow immunoassay. (**C**) The fluorescence analyzed by a smartphone and a fluorescence analyzer.

The optimization strategy was schematically described in Figure 1. EuPSM has excellent fluorescence characteristics and stability. Especially, the light collected by the detector could be not interfered by incident light due to the long Stokes shift. In addition, due to its larger size, EuPSM has a higher fluorescence intensity and can conjugate more probes (e.g., antibody). More probes bring more opportunities for binding with targets but may decrease response sensitivity. Therefore, we tried to optimize the probe density on EuPSM to construct ultra-sensitive LFIA. In order to eliminate the negative effect of non-specific binding sites, and to ensure the probes distributed uniformly, we introduced BSA as blank probes (Figure 1A).

The principle of the developed EuPSM-LFIA for CRP detection is achieved through the sandwich strategy, which is based on the sandwich structure of EuPSM-labeled anti-CRP Ab8#, target CRP, and anti-CRP Ab7#. Hence, the fluorescence intensity of the T-line gradually increases with the increasing concentration of the targets. The detection results can be read directly by a smartphone. To achieve quantitative analysis and improve detection sensitivity, a benchtop fluorescence analyzer was developed, which can build the relationship between fluorescence intensity and position of detection area through point-by-point scanning. As shown in the schematic (Figure 1C), the peak intensity at the T-line and C-line was analyzed.

### 3.2. Characterization of EuPSM

**Figure 2:**
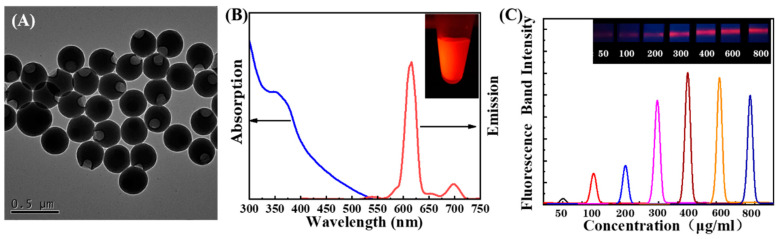
(**A**) TEM images of EuPSM. (**B**) Absorption spectrum and emission spectrum of EuPSM. (**C**) Estimation of probe saturation concentration on EuPSM.

Considering the limited pore size of the NC membrane used on LFIA, the EuPSM with a 300 nm diameter was finally selected. Uniform particle size and excellent monodispersity of EuPSMs are important for sensitive and stable detection. The transmission electron microscopy (TEM) image demonstrated that the prepared EuPSMs (~300 nm) are monodispersed with regular sphere structures (Figure 2A). From fluorescence spectrogram, the emission peaks at 610 nm were shown under 350 nm UV excitation due to long Stokes shift (Figure 2B). The inset shows that EuPSM emits strong red fluorescence under excitation light. These characteristics make EuPSM well suited for high-performance LFIA.

Since the probe density on the surface of EuPSM needs to be regulated, the saturated probe density on EuPSM needs to be predetermined. The saturated concentration of antibodies on EuPSM was estimated by the detection of a blank sample using LFIA. Anti-CRP 8# with different concentrations (50 μg/mL, 100 μg/mL, 200 μg/mL, 300 μg/mL, 400 μg/mL, 600 μg/mL, 800 μg/mL) were added to form UCNPs probes, where the binding ability was assessed by analyzing the C-line signal of the blank sample. Figure 2C shows that the fluorescence signals on the C-line increased with the concentrations of anti-CRP 8#; due to this, the binding ability increased with the number of probes on EuPSM. The fluorescence intensity remains unchanged when the concentration is above 400 μg/mL (2.7 nM), indicating that the probes modified on EuPSM reach saturation. 

### 3.3. Feasibility Detection of the LFIA

**Figure 3:**
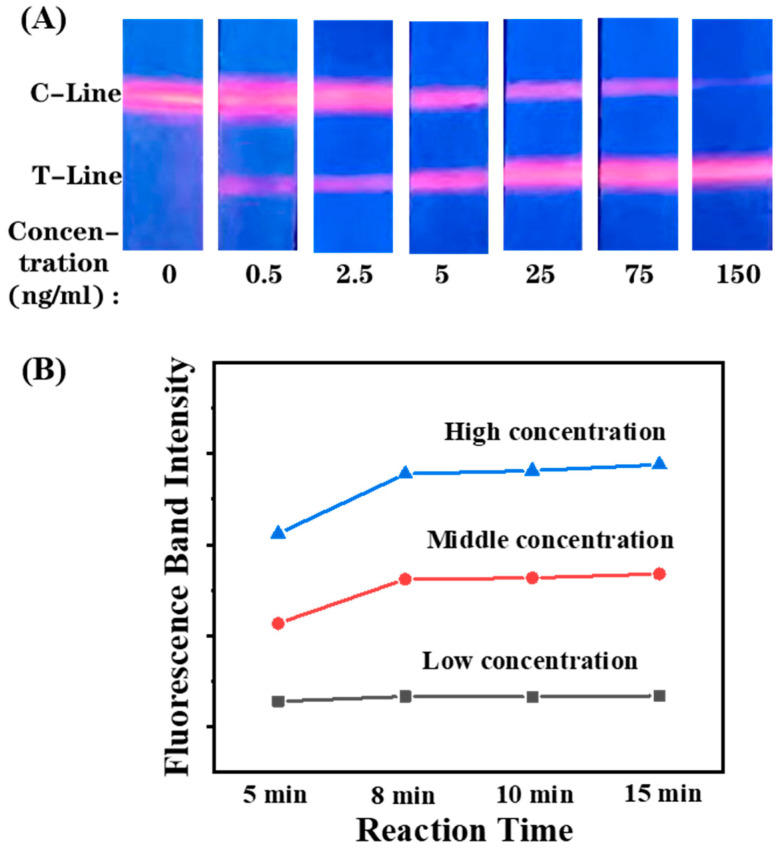
(**A**) The fluorescence photos of detecting gradient concentrations of CRP. (**B**) The relationship between fluorescence intensity and detection time.

After optimizations of the running buffer and antibody concentration on the T-line and C-line, the detection feasibility of the proposed UCNPs-LFIA was implemented by detecting CRP with gradient concentrations. As shown in Figure 3A, the developed EuPSM-LFIA has clear bands at the T-line and C-line in detection, showing low background interference. The fluorescence intensity of the T-line increased with the increasing concentration of CRP, and there are no false positive signals at the T-line at a negative detection, validating its detection feasibility. The signal can remain stable after 8 min, indicating that the developed platform can complete detection within 8 min (Figure 3B).

### 3.4. Optimization of Antibody Density on EuPSM

Although EuPSM with a large size allows the conjugation of more antibodies, the reduction of antibody density on the particle surface, to a certain extent, can effectively improve the detection sensitivity [26]; due to this, excessive antibodies could reduce the response between the fluorescence signal and sample concentration, thus decreasing detection sensitivity. Considering that directly reducing antibody density could cause uneven antibody modification, and the bare blank site could cause nonspecific binding, we tried to regulate the surface antibody density by introducing a blank probe.

**Figure 4:**
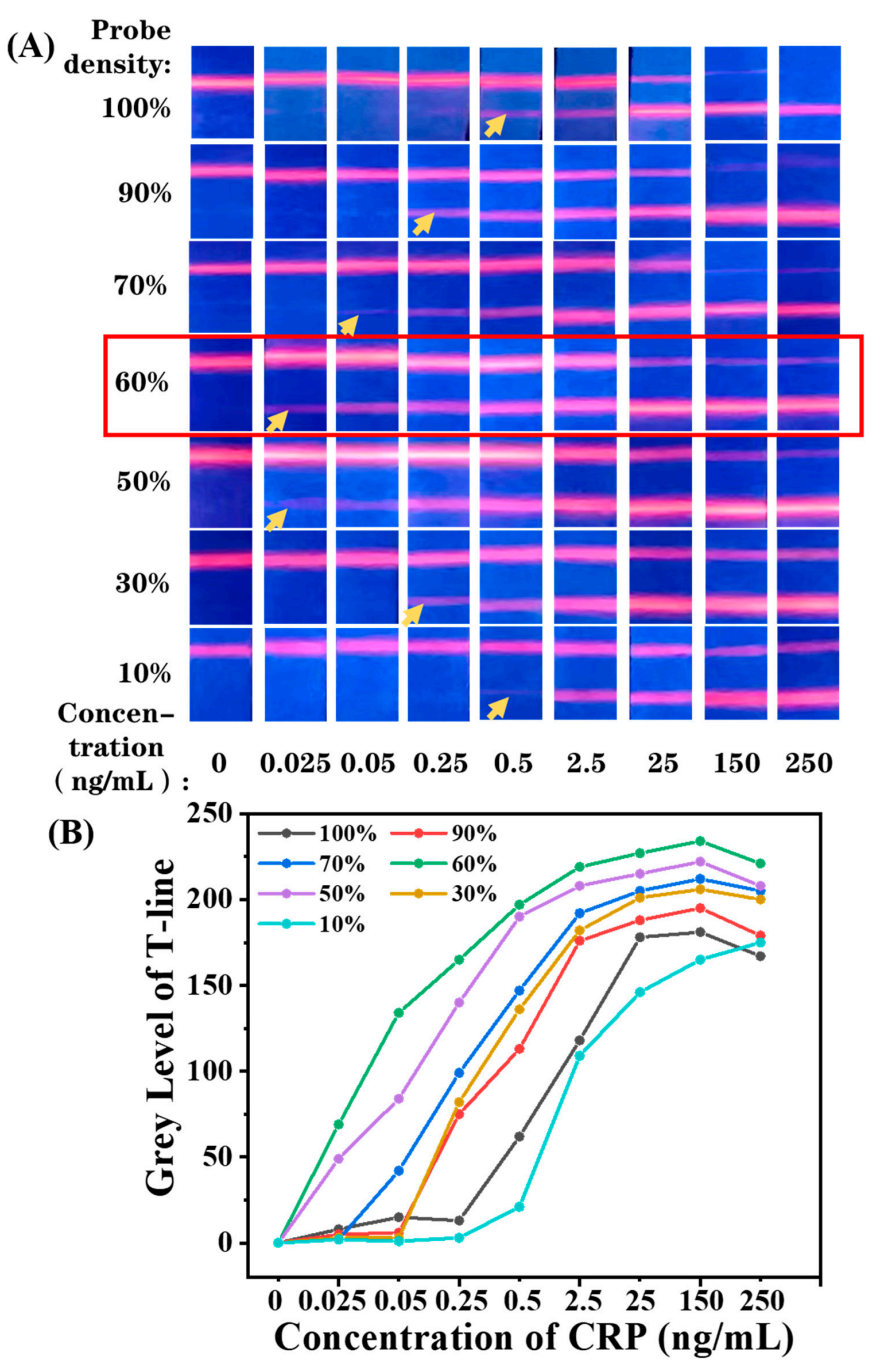
(**A**) Schematic illustration of antibody density affecting fluorescence signal at T-line and C-line. (**B**) The relationship curve of fluorescence intensity and CRP concentration with the different antibody density.

Therefore, to adjust the probe density on EuPSM, BSA was used as a blank probe and mixed with antibodies at different molar ratios to form a mixed probe with a total concentration of saturated probe concentration. The mixed probes were then conjugated with EuPSM to adjust the modified probe density. Based on this strategy, the antibody density on EuPSM was regulated to 90%, 70%, 60%, 50%, 30%, and 10% of the saturated density. The gradient concentrations of CRP standard samples were detected by EuPSM-based LFIA with a different probe density, and the fluorescence photos are shown in Figure 4A. 

Taking the group of 100% probe density as an example, the fluorescence photos show that the fluorescence band was enhanced gradually at the T-line and faded at the C-line with the increase in the CRP concentration. The T-line showed a weak fluorescence band at the concentration of 0.5 ng/mL and no fluorescence signal at lower level, which means that the lowest distinguishable concentration by the naked eye can reach 0.5 ng/mL. In addition, due to the strong binding ability of particles with high probe density, they are more likely to be captured by the T-line, so that the fluorescence signal on C-line decreases with the increase in CRP concentration. As shown in Figure 4A, the fluorescence band of the C-line disappeared at the concentration of 150 ng/mL, which was identified as the highest valid concentration, as the absence of the fluorescence band at the C-line is usually considered as a sign of LFIA failure. With the antibody density decreasing from 100% to 60%, the lowest distinguishable concentration decreased gradually and achieved 0.025 ng/mL at 60% probe density. When the probe density is below 60%, the lowest distinguishable concentration starts to rise again, which returned to 0.5 ng/mL at 10% probe density. The main reason for this is that the fluorescence signal is determined by both response sensitivity and binding capacity. When the antibody density was lower than 60%, the EuPSM-anti-CRP Ab8# was not easily captured by anti-CRP Ab7# at the T-line because of low binding capacity, resulting in reduced fluorescence signal. In short, antibody density on the surface of particles not only affects the response of fluorescence signals to biological signals, but also affects the binding capacity. With the antibody density decreasing, the detection sensitivity first decreases and then increases, reaching the best value at 60% of antibody density. In order to make the results more intuitive, we extracted the average gray value of the T-line and drew the relationship curve. As shown in Figure 4B, the grey level of 60% probe density changed earlier with the increased concentration, and its fluorescence intensity was also highest, indicating that its detection performance is best. In addition, with the decrease in antibody density, the C-line signal gradually decreases with the increase in the target concentration, which can effectively improve the detection range of the detection platform. 

### 3.5. Optimization of EuPSM-Antibody Concentration

**Figure 5:**
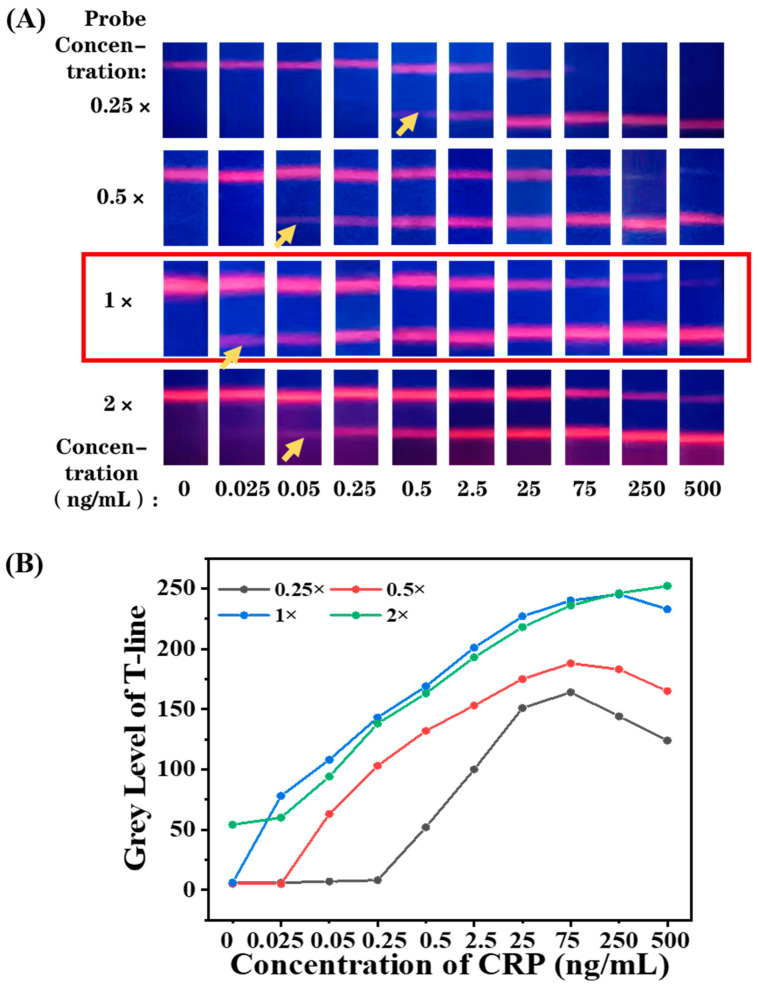
(**A**) The fluorescence photos of LFIA with different EuPSM-antibody concentration. (**B**) The relationship curve of fluorescence band intensity and CRP concentration with different EuPSM-antibody concentrations.

In the above experiments, the antibody density on EuPSM was adjusted to optimize the response between fluorescence signals and biological signals. Next, we continued to optimize the concentration of EuPSM-anti-CRP Ab8# to further optimize the detection performance of EuPSM-LFIA. Different concentrations (2×, 1×, 0.5×, and 0.25×) of EuPSM-anti-CRP Ab8# (60% probe density, 0.5 mg/mL of original concentration) were studied. The fluorescence photos and their quantization curves are shown in Figure 5A,B. We found that under low concentration (0.25×), the fluorescence signal on both the T-line and C-line was very weak, resulting in the detection range of 0.5–25 ng/mL. With the increase in EuPSM-anti-CRP Ab8# concentration, the fluorescence signal was greatly enhanced. Although the 2× concentration group showed a significant increase in the upper detection limit (>500 ng/mL), the high probe concentration will significantly increase the background color, posing difficulties in how to distinguish the low concentration target from the background. The 1× probe concentration showed the optimal detection range (0.025–250 ng/mL). 

### 3.6. Development of a Benchtop Fluorescence Analyzer

**Figure 6:**
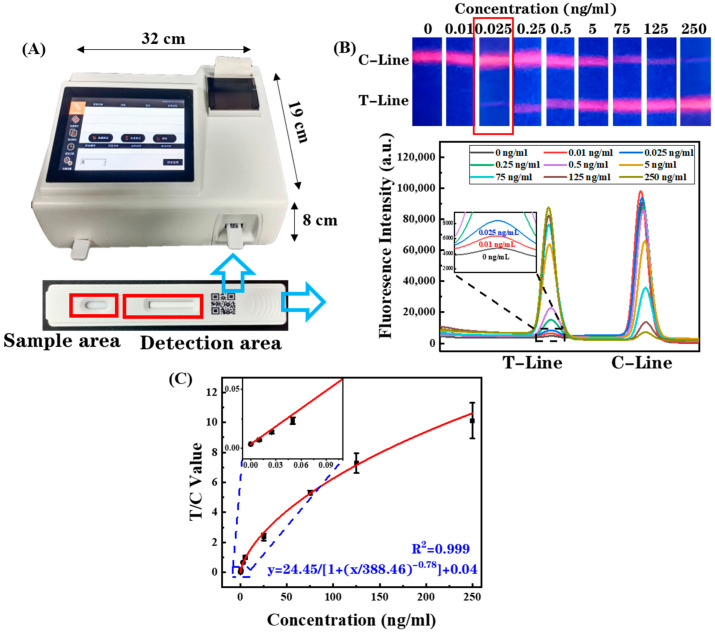
(**A**) Real products of the benchtop fluorescence analyzer. (**B**) The detection results read by the benchtop fluorescence analyzer compared with fluorescence photos. (**C**) The standard curve of CRP detection analyzed by the benchtop fluorescence analyzer.

A benchtop fluorescence analyzer was developed to realize the automated quantification of the results of EuPSM-LFIA (Figure 6A). Through the built-in 350 nm point laser, the detection area of the inserted EuPSM-LFIA was scanned point by point. The average fluorescence intensity was collected, forming a graph of the relationship between fluorescence intensity and scanning position. The peak height and position correspond to the fluorescence signal of the T-line and C-line. It can be observed from the fluorescence graph that the fluorescence intensity of the T-line increased and the C-line decreased, with the increasing concentration of CRP (Figure 6B), which corresponds to the fluorescence photos by the smartphone. Although the fluorescence band could only be distinguished at the concentration of 0.05 ng/mL through the naked eye, the analyzer still can monitor obvious signal changes at lower concentrations (0.01 ng/mL), which indicates that the detection sensitivity can be improved through the benchtop fluorescence analyzer. Similarly, the previously unobservable C-line signal (as shown at 250 ng/mL) can also obtain a weak signal from the analyzer, which indicates that the larger detection range can be analyzed.

Particularly, since the fluorescence signal at the C-line changes with different target concentrations, the fluorescence intensity ratio of the T-line and C-line (T/C) corresponding to target concentrations showed a good upward trend. The use of the T/C value as the detection signal not only amplifies the range of signal variation, but also effectively improves the lower detection limit and upper detection limit. Standard curves of the T/C value and CRP concentration were plotted according to four parameters. As shown in Figure 6C, the fitted curves with a high correlation coefficient (R^2^ = 0.999) were obtained, showing high accordance with the detection results of the fluorescence photos. Furthermore, the analytic sensitivity (T/C value/concentration) decreases with the increase in concentration. 

Considering that the systematic error can directly affect the value of the LOD [27], the blank samples (0 ng/mL) were detected 10 times. An average T/C value of 0.00354 ± 0.00009047 and a coefficient variation (CV) of 2.5% were obtained, proving that the systematic error is very low. The LOD of 0.76 pg/mL was obtained, which was calculated referring to the LOD calculation method in the literature [17,18] (blank signal + three standard deviations). The upper detection limit was 250 ng/mL, which was determined according to the minimum analytic sensitivity. Through comparison, the developed platform showed greater advantages to other POCT methods (Table 1). In addition, it has been proven by repeated reading experiments that the results deviation of reading the same samples using the analyzer is less than 2%, which proves the excellent reliability of the analyzer. 

### 3.7. Clinical Application

**Figure 7:**
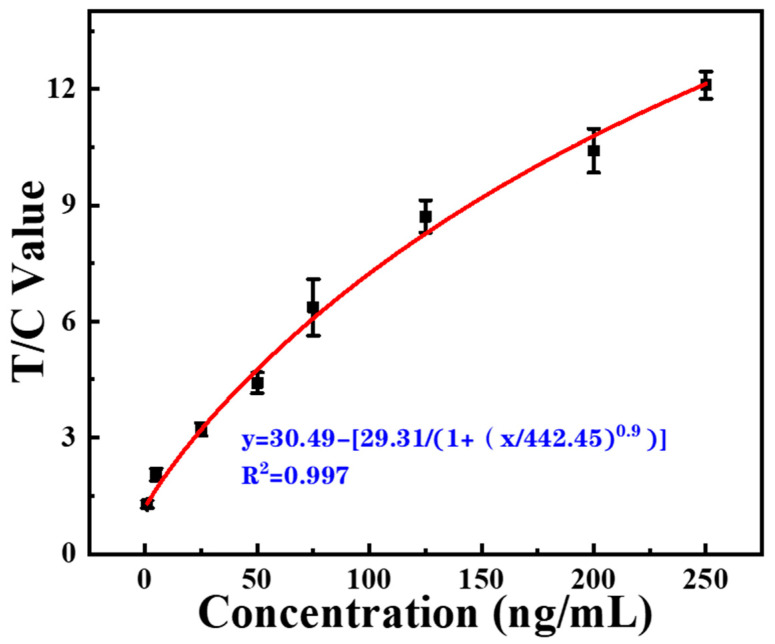
Standard curve of optimized EuPSM-LFIA for CRP detection in clinical serum sample.

To assess the practical application ability of developed EuPSM-LFIA, we measured the concentration of CRP in clinical serum samples using the developed platform and compared the detection results with standard clinical detection results (obtained by the chemiluminescent immunoassay method). Standard serum samples were prepared by adding CRP with gradient concentrations, and were further used to draw a standard curve (Figure 7). Based on this curve, 11 serum samples were diluted 500 times using the running buffer. The obtained T/C values were introduced to the standard curve to calculate the concentration. The calculated concentration and standard concentration were recorded in Table 2. All the detected results were in the area between the two dotted lines (±15%), which indicated that the developed EuPSM-LFIA shows no significant difference with those of clinical methods and meets the requirements for clinical use. All these results can prove that the developed EuPSM-LFIA platform can be effectively applied to clinical application. It is worth emphasizing that the cost of developing LFIA platform is low, where a single LFIA (3 Yuan RMB) and a reusable ultraviolet excitation light (10 Yuan RMB) are needed for rapid, qualitative testing. If quantitative detection is required, an additional bench fluorescence analyzer (1000 Yuan RMB) can be purchased. The average cost is much lower than that of clinical testing methods in hospital (80 Yuan RMB per test)

## 4. Conclusions

In this paper, we developed an EuPSM-based LFIA integrated with a benchtop fluorescence analyzer for hs-CRP detection. The performance of the EuPSM-based LFIA was optimized through adjusting the antibody density on EuPSM. Finally, 60% of the saturated density was verified to show the best performance. Through further optimizing, the concentration of EuPSM-anti-CRP Ab8#, the LOD of 0.76 pg/mL, and detection range of 0.025–250 ng/mL were obtained. Noteworthily, the clinical application capability of the proposed POCT platform was validated through detecting CRP in clinical serum samples, which showed a high consistency with the results obtained from the clinical standard method. Hence, the proposed EuPSM-based LFIA was verified to be well suited for hs-CRP detection with ultra-high detection sensitivity and wide detection range, while also showing huge potential for sensitively and rapidly detecting other biomarkers.

## Figures and Tables

**Table 1:** Comparison of detection performance through different POCT methods for CRP detection.

Methods	LOD (ng/mL)	Upper Limit (ng/mL)	Detection Time (min)	Reference
Fluorescent-based LFA	91	1.6 × 10^5^	3	[28]
Fluorescent-based LFA	3.89	1.6 × 10^3^	30	[29]
QDs-based LFA	0.3	10^3^	3	[18]
QDs-based LFA	0.25	300	15	[30]
SERS based LFA	0.01	10^3^	unknown	[31]
Core-shell modified UCNPs-LFA	0.05	50	8	[19]
Magnetic chemiluminescent immunoassay	1.5	81	30	[32]
QDs-based immunosorbent assay	0.45	400	50	[33]
Probe density regulated EuPSM-LFA	0.00076	250	8	Our work

**Table 2:** Comparison of detection value and actual concentration.

Numbering	Actual Concentration (ng/mL)	Detection Concentration (ng/mL)	Deviation (SD/M)
1	21.3	19.98	6.19%
2	34.6	33.45	1.80%
3	7.22	6.62	8.30%
4	70.16	64.75	7.71%
5	9.96	8.85	11.15%
6	91.12	77.57	14.87%
7	119.9	111.21	7.24%
8	12.76	11.62	8.99%
9	17.78	17.73	0.28%
10	213.9	231.62	8.28%
11	243.12	239.44	1.51%

## Data Availability

Not applicable.
